# PAS staining of bronchoalveolar lavage cells for differential diagnosis of interstital lung disease

**DOI:** 10.1186/1746-1596-4-13

**Published:** 2009-04-23

**Authors:** Hans P Hauber, Peter Zabel

**Affiliations:** 1 Division of Pathophysiology of Inflammation, Experimental Pneumology, Research Center Borstel, Germany

## Abstract

Bronchoalveolar lavage (BAL) is a useful diagnostic tool in interstitial lunge diseases (ILD). However, differential cell counts are often non specific and immunocytochemistry is time consuming. Staining of glyoproteins by periodic acid Schiff (PAS) reaction may help in discriminating different forms of ILD. In addition, PAS staining is easy to perform. BAL cells from patients with idiopathic pulmonary fibrosis (IPF) (n = 8), sarcoidosis (n = 9), and extrinsic allergic alveolitis (EAA) (n = 2) were investigated. Cytospins from BAL cells were made and cells were stained using Hemacolor quick stain and PAS staining. Lymphocytic alveolitis was found in sarcoidosis and EAA whereas in IPF both lymphocytes and neutrophils were increased. PAS positive cells were significantly decreased in EAA compared to IPF and sarcoidosis (25.5% ± 0.7% vs 59.8% ± 25.1% and 64.0% ± 19.7%, respectively) (P < 0.05). No significant correlation between PAS positive cells and inflammatory cells was observed. These results suggest that PAS staining of BAL cells may provide additional information in the differential diagnosis of ILD. Further studies ware warranted to evaluate PAS staining in larger numbers of BAL from patients with ILD.

## Findings

Bronchoalveolar lavage (BAL) provides an important diagnostic tool that can facilitate the diagnosis of various diffuse lung diseases. It can be used to determine inflammatory cell profiles and detect pathogens [1]. BAL cytology and differential cell counts can even replace histology from lung biopsies in some of the rarer lung diseases (eg. histiocytosis X) [2]. However, in more common interstitial lung disease (ILD) such as idiopathic pulmonary fibrosis (IPF), sarcoidosis, and extrinsic allergic alveolitis (EAA) BAL differential cell counts often cannot clearly differentiate between the different disorders. Immuncytochemistry can provide additional information but is time consuming. Moreover, specific antibodies are expensive and are not available at all places.

PAS (periodic-acid-Schiff) staining is used for detection of structures that contain high concentrations of carbohydrate macromolecules (eg. glycogen, glycoprotein, proteoglycan) typically found in connective tissue, mucus, and basal laminae. In BAL PAS staining is mostly used to diagnose alveolar proteinosis [2]. In ILD increased BAL fluid levels of the glycoprotein fibronectin have been reported [3]. Elevated levels of vitronectin another glycoprotein have also been shown in BAL fluid of patients with interstitial lung disease compared to healthy volunteers [4]. Since PAL stain is an easy method to detect glycoproteins we hypothesized that PAS stain may add additional information for differential diagnosis of ILD from BAL.

BAL samples of patients with proven idiopathic pulmonary fibrosis (IPF) (n = 8), with sarcoidosis (n = 9), and with extrinsic allergic alveolitis (EAA) (n = 2) were obtained with the help of flexible bronchoscopy. Table 1 shows the demographics of the patient groups.

**Table 1:** Patient group demographics

Group	IPF **(**N = 8**)**	Sarcoidosis (N = 9)	EAA (N = 2)
Male:female	6:2	7:2	0:2

Mean age (years)	67.4 ± 9.9	45.4 ± 14.8	57.0 ± 19.8

Age values are given as mean ± SD

In short, a total of 200 ml of sterile saline solution was instilled in 20 ml-aliquots into the middle lobe and aspirated thereafter. Following standard techniques [5] cytospins were made from BAL cells and cells were stained using Hemacolor quick stain (Merck, Darmstadt, Germany) and PAS staining. Table 2 summarizes the differential cell counts in each patient group. As expected lymphocytic alveolitis could be observed in all groups. Lymphocytes were significantly increased in patients with EAA compared to the groups with IPF or sarcoidosis (P < 0.05). Neutrophils were significantly increased in IPF patients compared to the two other groups (P < 0.05).

**Table 2:** Differential BAL cell counts in different patient groups

Group	AM (%)	Lymphocytes (%)	Neutrophils (%)	Eosinophils (%)
Pulmonary fibrosis	37.8 ± 14.7	12.4 ± 7.5	12.5 ± 13.9	1.6 ± 1.9

Sarcoidosis	70.0 ± 12.5	26.6 ± 13.9	2.2 ± 2.6	1.3 ± 1.2

EAA	7.0 ± 2.8	86.5 ± 0.7	3.0 ± 1.4	0.5 ± 0.7

AM: Alveolar macrophages. Mean ± SD

**Figure 1:**
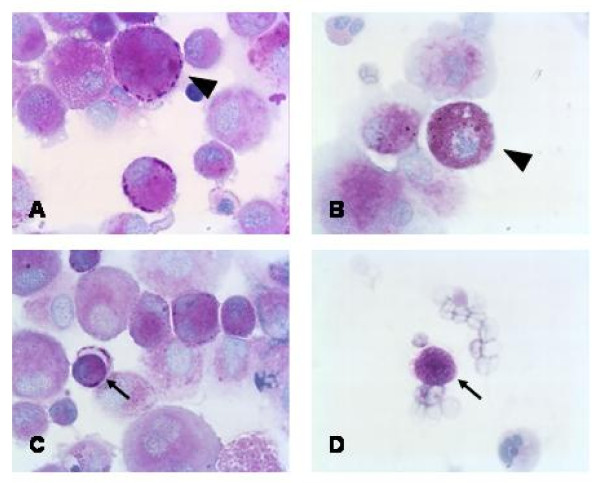
**Original microscopy of PAS stained BAL cells (magnification ×400)**. Alveolar macrophages (shown by arrow heads in figures 1A and 1B) as well as lymphocytes (indicated by arrows in figures 1B and 1C) stained positive (pink).

**Figure 2:**
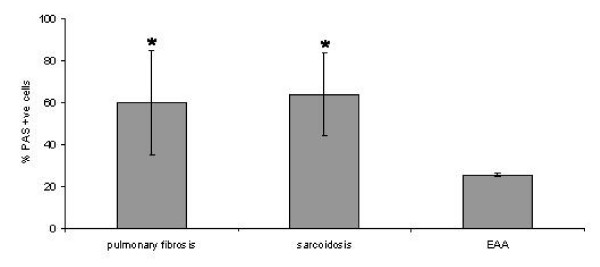
**PAS positive cells in different patient groups**. Columns represent mean values ± SD. For abbreviations please refer to the text. *: P < 0.05 vs EAA.

Figure 1 shows original PAS stained cytospins. Alveolar macrophages (Figure 1A, B) as well as lymphocytes (Figure 1C, D) stained PAS positive. The numbers of PAS positive BAL cells from patients with EAA were significantly lower compared to patients with IPF or sarcoidosis (25.5% ± 0.7% vs 59.8% ± 25.1% and 64.0% ± 19.7%, respectively) (P < 0.05) (Figure 2). No significant correlation between the numbers of PAS positive cells and any kind of inflammatory cells in the BAL was observed.

The demographics of the groups show that patients with IPF and EAA were markedly older than patients with sarcoidosis. This is not unusual in clinical practice since sarcoidosis is more often observed in younger people and IPF is typically found in older patients [6,7]. Therefore the patient groups seem to be representative for the age and disease.

The findings in the present study demonstrated a marked decrease in PAS positive BAL cells in patients with EAA compared to other interstitial lung diseases such as IPF and sarcoidosis. However, we only investigated a very small number of EAA patients but both patients had a low percentage of PAS positive BAL cells (25% and 26%). This analysis of individual data suggests that in PAS positivity of BAL cells may be in fact reduced in EAA but this has to be further investigated with larger numbers of samples.

We did not find a significant correlation between PAS staining and a single cell type and I did not do double staining to differentiate PAS positive cells. Therefore it is not clear which inflammatory cells mostly stained positive. Future studies will have to identify whether macrophages, lymphocytes or neutrophils are the main source of PAS positive material. Another option is that PAS positive proteins undergo phagocytosis by alveolar macrophages. In fact, several proteins (eg. surfactant proteins) are secreted by pneumocytes [8]. These proteins have different functions and are also involved in pulmonary defense. Moroever, they can opsonize pathogens and support phagocytosis. In the original microscopy in figure 1 both macrophages and lymphocytes stained PAS positive. The staining was diffuse in some cells whereas a more granular pattern was observed in other cells. The reason for this remains unclear. It is tempting to speculate that specific staining pattern may be associated with different glycoproteins and different stages of disease. However, the numbers examined in this study are too small to draw any firm conclusions.

In the present study IPF, sarcoidosis and EAA were not further subdivided into different stages of disease due to the small numbers of patients. It may be interesting to look whether percentage of PAS positive cells is associated with different stages of disease (eg. active alveolitis versus fibrosis). However, this was beyond the scope of the present investigation.

So far no experiments on PAS staining in other interstitial lung disease such as pneumoconiosis or Langerhans cell histiocytosis were performed but these studies would clearly be very helpful to further evaluate the role of PAS staining in discrimination of ILD. Own experiments on patients with allergic bronchopulmonary aspergillosis (ABPA) and pneumonia revealed a mean of PAS positive cells of 70.5% and 47.5%, respectively. It is interesting that in pneumonia that is commonly associated with mucus hypersecretion the mean rate of PAS positive cells was lower than in BAL cells from patients with sarcoidosis or IPF. This further underlines the fact that PAS staining identifies glycoproteins and not only mucin proteins.

In conclusion the present findings suggest that PAS stain of BAL cells may be helpful in differential diagnosis between EAA and other ILD. Although the numbers of patients investigated in this study was very small we think that further studies are warranted to evaluate the use of PAS stain in different forms of ILD. Should future studies prove that PAS can discriminate different ILD then this would be a very easy and less expensive tool for BAL-based differential diagnosis.

## Abbreviations

BAL: bronchoalveolar lavage; EAA: extrinsic allergic alveolitis; IPF: idiopathic pulmonary fibrosis; PAS: periodic acid Schiff

## Competing interests

The authors declare that they have no competing interests.

## Authors' contributions

HPH planned and performed the experiments. HPH and PZ did the data analysis and wrote the manuscript.
